# Community Structure and Predicted Functions of Actively Growing Bacteria Responsive to Released Coral Mucus in Surrounding Seawater

**DOI:** 10.1264/jsme2.ME23024

**Published:** 2023-09-14

**Authors:** Akito Taniguchi, Yuki Kuroyanagi, Ryuichiro Aoki, Mitsuru Eguchi

**Affiliations:** 1 Faculty of Agriculture, Kindai University, 3327-204 Naka-machi, Nara, Nara 631-8505, Japan; 2 Graduate School of Agriculture, Kindai University, 3327-204 Naka-machi, Nara, Nara 631-8505, Japan; 3 Agricultural Technology and Innovation Research Institute, Kindai University, 3327-204 Naka-machi, Nara, Nara 631-8505, Japan

**Keywords:** coral mucus, bromodeoxyuridine, actively growing bacteria, community structure, function

## Abstract

A direct relationship exists between diverse corals and fish farming in Keten Bay, Amami-Oshima, Japan. The release of coral mucus has a significant impact on the microbial activity of surrounding seawater. To obtain a more detailed understanding of biogeochemical cycles in this environment, the effects of coral mucus on the community structure and function of bacteria in surrounding seawater need to be elucidated. We herein used a bromodeoxyuridine approach to investigate the structures and functions of bacterial communities growing close to mucus derived from two different *Acropora* corals, AC1 and AC2. The alpha diversities of actively growing bacteria (AGB) were lower in mucus-containing seawater than in control seawater and their community structures significantly differed, suggesting that the growth of specific bacteria was modulated by coral mucus. *Rhodobacteraceae* and *Cryomorphaceae* species were the most dominant AGB in response to the mucus of *Acropora* AC1 and AC2, respectively. In contrast, the growth of *Actinomarinaceae*, *Alteromonadaceae*, *Flavobacteriaceae*, and SAR86 clade bacteria was inhibited by coral mucus. The results of a Phylogenetic Investigation of Communities by Reconstruction of Unobserved States (PICRUSt2) ana­lysis suggested that the predicted functions of AGB in mucus-containing seawater differed from those in seawater. These functions were related to the biosynthesis and degradation of the constituents of coral mucus, such as polysaccharides, sugar acids, and aromatic compounds. The present study demonstrated that complex bacterial community structures and functions may be shaped by coral mucus, suggesting that corals foster diverse bacterial communities that enhance the ecological resilience of this fish farming area.

Mucus released into seawater by corals has a significant impact on neighboring microbes. Corals produce mucus comprising a mucopolysaccharide glycoprotein lipid complex (Brown and Bythell, 2005; Bythell and Wild, 2011) with amino acids, carbohydrates, and inorganic nutrients (Wild *et al.*, 2005; Nakajima *et al.*, 2009, 2015; Tanaka *et al.*, 2009). More than half of coral mucus exists in the dissolved fraction (Nakajima *et al.*, 2010), while the remaining fractions form aggregates, such as strings, webs, and/or sheets (Naumann *et al.*, 2009; Bythell and Wild, 2011). Mucus also contains a holobiont or a surrounding community of microbes, such as bacteria and protozoa (Rohwer *et al.*, 2002). Since microbial responses and processes may be affected at the microscale (Azam, 1998), the impact of coral mucus on holobiont dynamics cannot be ignored.

Previous studies demonstrated that coral mucus modulated the microbial activity of surrounding seawater. Some of the most prominent effects of coral mucus on microbial activity include markedly higher microbial abundance (Ferrier-Pagès *et al.*, 2000; Nakajima *et al.*, 2017), production (Taniguchi *et al.*, 2014), and respiration (Wild *et al.*, 2004) than in non-mucus-containing seawater. Taniguchi *et al.* (2014) showed that even a 1:3,000 mucus:seawater ratio (v/v) was sufficient to enhance bacterial production by approximately 2-fold from that by unsupplemented seawater. However, coral mucus and its associated bacteria may also exhibit antimicrobial properties to select beneficial microbes for corals (Ritchie, 2006; Shnit-Orland and Kushmaro, 2009). Ritchie (2006) demonstrated that coral mucus inhibited bacterial growth by up to 10-fold.

These characteristics of coral mucus also have a significant impact on the community structures of bacteria in surrounding seawater. Allers *et al.* (2008) showed that bacteria belonging to *Gammaproteobacteria*, particularly *Alteromonadaceae* and *Vibrionaceae*, became dominant after the incubation of seawater with *Fungia* mucus at a 1:10 mucus:seawater ratio (v/v). Taniguchi *et al.* (2015) reported a marked shift in bacterial community structures in‍ ‍*Acropora* mucus-supplemented seawater (1:300) over a‍ ‍10-‍h‍ ‍incubation and that *Rhodobacterales* bacteria (*Alphaproteobacteria*) became dominant in the late phase of the incubation. However, the denaturing gradient gel electrophoresis method used in that study had limitations for the detection of bacteria due to its moderate resolution of bacterial species and its ability to identify only a small number of species. Furthermore, previous studies collected coral mucus by exposing corals to air, which may have resulted in differences in the composition of the mucus collected from that of naturally released mucus underwater (Brown and Bythell, 2005). In addition to enhancing bacterial growth, coral mucus exhibits antimicrobial activity against pathogens (Shnit-Orland and Kushmaro, 2009; Ritchie, 2006), which needs to be considered when examining the overall effect of coral mucus on microbial communities. The responses of bacterial community structures to coral mucus have also been shown to vary among coral species (Nelson *et al.*, 2013) and coral stress levels (Lee *et al.*, 2016), suggesting that coral mucus forms complex microbial ecosystems in coral reef environments.

Cage cultures for fishery species, such as bluefin tuna (*Thunnus orientalis*), red seabream (*Pagrus major*), and white trevally (*Pseudocaranx dentex*), have been pursued in Keten Bay, Amami-Oshima, Japan for approximately 20 years. Among the wide variety of corals inhabiting the cages and ropes, species of the genus *Acropora* corals are the largest colonizing corals (Hata *et al.*, 2013). *Acropora* species represent the dominant hard corals in the Great Barrier Reef, where they reportedly release 4.8 L m^–2^ d^–1^ of mucus containing 56–80% of the dissolved fraction (Wild *et al.*, 2004). The effects of released coral mucus on the bacterial community and its functions in surrounding seawater remain unclear. Therefore, the effects of mucus produced by *Acropora* corals on bacterial community structures and functions warrant further study.

We herein examined the community structure of bacteria, particularly actively growing bacteria (AGB), in seawater containing *Acropora* mucus obtained by nondestructive sampling, which minimizes stress on the coral. We used bromodeoxyuridine (BrdU) as a DNA tracer to assess the AGB community structure and its function and identified bacterial communities that responded to a coral mucus stimulation and investigated their functions. The results obtained herein showed that coral mucus led to the dominance of specific bacteria, such as *Rhodobacteraceae* and *Cryomorphaceae*, and the occurrence of distinct functions according to mucus composition, which affected bacterial activities and shaped their complex community structure and functions in the neighboring seawater environment.

## Materials and Methods

### Sample collection

Seawater and coral mucus were collected between 2010 and 2013 in Keten Bay, Amai-Oshima, Kagoshima, Japan. Healthy corals on the ropes of Pacific bluefin tuna culture cages (N28.223, E129.216) were used: four samples of *Acropora* sp. AC1 between October 2010 and May 2012 and two samples of *Acropora* sp. AC2 between October 2012 and May 2013 (Fig. S1). Although *Acropora* species were differentiated based on their appearance (Fig. S1), they may have belonged to the same species. Mucus was collected non-destructively from seawater near the coral surface (within 1‍ ‍cm) using a sterile syringe. Surface seawater was taken from the mouth of Keten Bay (N 28.2192, E 129.2171), far from the corals. Three treatment conditions were prepared, each in duplicate: the original coral mucus (100% coral mucus, MuSW100), a 1:1 mixture of seawater and coral mucus (50% coral mucus, MuSW50), and the seawater (0% coral mucus, SW). Five liters of water from each condition was treated with BrdU (final concentration 1‍ ‍μM) and incubated for 10‍ ‍h at an ambient seawater temperature (Table S1) in the dark to avoid DNA damage caused by the light sensitivity of cells due to BrdU incorporation. Before (T0, 250–1,000‍ ‍mL) and after the incubation (T10, ~3,000‍ ‍mL), bacterial cells were collected onto a Streivex cartridge filter with a pore size of 0.22‍ ‍μm (Millipore) using a peristaltic pump (Fig. S2), and the filter was frozen and stored until further ana­lyses. Environmental parameters, such as water temperature, salinity, the concentration of chlo­rophyll *a* (Chl *a*), and turbidity, were measured using a modernized, compact, and lightweight multiparameter water quality meter (AAQ175; JFE Advantech), and seawater transparency was assessed using a Secchi disk (Rigo). Total organic carbon (TOC) concentrations were measured with a TOC analyzer (TOC-V CPH, Shimadzu).

### Total and BrdU-incorporated bacterial community structures

DNA extraction was performed according to previously described procedures (Hamasaki *et al.*, 2007) with a slight modification; we removed the filter housing and performed three rounds of incubation using xanthogenate sodium dodecyl sulfate buffer (a cell lysis step) to increase the recovery rate of DNA. The immunocapture of BrdU-labeled DNA from 1,000‍ ‍ng of extracted total DNA was performed according to previously described procedures (Hamasaki *et al.*, 2007). DNA from a sample without the BrdU incubation was used as a negative control to confirm successful immunocapture. We defined sample DNA before and after immunocapture as total (T0 and T10) and the BrdU-labeled fraction, respectively. After this validation, total and BrdU-labeled DNA samples were subjected to an automated ribosomal fragment analysis (ARISA) according to previously described methods (Fuhrman *et al.*, 2006; Taniguchi *et al.*, 2022) to assess the level of variation between bottles. Illumina MiSeq sequencing for selected samples was performed by Hokkaido System Science, which focused on a 300-bp paired-end sequence (301 cycles×2) for the V3–V4 region of 16S rRNA (341F forward, 5′-CCTACGGGNGGCWGCAG-3′; 805R reverse, 5′-GACTACHVGGGTATCTAATCC-3′) (Herlemann *et al.*, 2011) targeting both bacteria and archaea.

### Sequence processing and statistical ana­lysis

Sequenced reads were analyzed using the Qiime2 Core 2021.2 distribution (Bolyen *et al.*, 2019). Raw sequence data were demultiplexed using the q2-demux plugin and the removal of primer sequences, low-quality sequences (Q<20), and chimeric reads were performed using DADA2 (Callahan *et al.*, 2016). Taxonomy assignment to amplicon sequence variants (ASVs) was conducted using the q2‐feature‐classifier plugin (Bokulich *et al.*, 2018) of the classify‐sklearn naïve Bayes taxonomy classifier against the Silva database (silva-138-99). ASVs related to mitochondria, chloroplasts, Cyanobacteria, and unassigned taxa were removed from further ana­lyses. All ASVs aligned with mafft (Katoh *et al.*, 2002) via the q2‐alignment were used for phylogenic tree construction with fasttree2 (Price *et al.*, 2010) via q2‐phylogeny. Functions based on MetaCyc (Caspi *et al.*, 2013) and Enzyme Commission (EC) number databases were predicted with ASVs using a Phylogenetic Investigation of Communities by Reconstruction of Unobserved States (PICRUSt2) ana­lysis (Douglas *et al.*, 2020) via q2-picrust2. Alpha‐diversity metrics (observed distinct microbial features, Faith’s phylogenetic diversity only for ASV, Shannon entropy, and Pielou’s Evenness) were computed after rarefication with minimum sequences or abundance for ASV or functions predicted by PICRUSt2 using q2-diversity. A beta-diversity Bray-Curtis dissimilarity index for bacterial community structures was calculated with normalized relative abundance per sample using q2-diversity, and results were subjected to a principal coordinate ana­lysis (PCoA).

All statistical ana­lyses were performed using R software version 4.2.2 (R Core Team, 2022). To analyze microbial compositions and functions, we processed the ASV table using the “phyloseq” package (McMurdie and Holmes, 2013). Tukey’s honestly significant difference (HSD) test was performed to examine the effects of coral mucus carbon using the “multcomp” package (Hothorn *et al.*, 2016). Clusters based on Bray-Curtis dissimilarity and Jaccard indices, which were calculated based on ARISA peak patterns, were constructed with the between-group average linkage method using the “vegan” package (Oksanen *et al.*, 2022). To distinguish significant clusters, a similarity profile routine (SIMPROF) with 9999 permutations was performed using the “clustsig” package (Whitaker and Christman, 2014). A comparison of alpha diversities between communities of total (T0) and BrdU-labeled microbes in each sample was conducted using the Wilcoxon signed-rank test with the “exactRankTests” package (Hothorn and Hornik, 2022). To test for differences in the relative abundance of bacterial taxa among the SW, MuSW50, and MuSW100 samples, the Kruskal-Wallis rank sum test was performed and followed by the Wilcoxon signed-rank test with a Benjamini-Hochberg correction (Benjamini and Hochberg, 1995). To examine differences in microbial community structures, we performed PERMANOVA with 10,000 permutations using the adonis2 function with the “vegan” package. Differentially abundant microbial taxa and functions with a log2 fold change (log2FoldChange)>|1| and adjusted *P*<0.01 were estimated between SW and MuSW100 samples using the “DESeq2” package (Love *et al.*, 2014) with the default parameters.

### Accession number of raw sequence data

Raw sequence data obtained in the present study have been deposited in the DNA Data Bank of Japan (DDBJ) (https://www.ddbj.nig.ac.jp/) Sequence Read Archive under the accession number DRA015913.

## Results

### Environmental characteristics

During the sampling period, water temperature and salinity were in the ranges of 22.78–27.44°C and 34.11–34.56‍ ‍psu, respectively (Table S1), and Chl *a* concentrations ranged between 0.27 and 0.79‍ ‍μg L^–1^. TOC concentrations in MuSW100 and MuSW50 samples ranged between 0.931 and 2.771‍ ‍mg L^–1^, with averages (±SD) of 1.760±0.621‍ ‍mg‍ ‍L^–1^ and 0.868–1.787 (1.249±0.349) mg L^–1^, respectively. TOC concentrations in SW samples ranged between 0.792 and 1.349‍ ‍mg L^–1^, with an average of 0.947±0.162‍ ‍mg L^–1^ (Table 1, Tukey-HSD *P*<0.05). The percentage of carbon derived from coral mucus ranged between 2.8 and 70.7% in MuSW50 samples and between 10.3 and 155.4% in MuSW100 samples.

### Community structures of total and BrdU-labeled bacteria by ARISA

A cluster ana­lysis of the ARISA peak pattern of the internal transcribed spacer region from total DNA showed the differential community structures of both total and BrdU-labeled bacteria between SW and mucus-containing SW (MuSW50 and MuSW100) samples (SIMPROF, *P*<0.05) (Fig. S3). In MuSW50 and MuSW100 samples, the bacterial community structures of T0 (before incubation) samples were distinct from those of T10 (after a 10-h incubation) samples. Furthermore, different clusters formed between total and BrdU-labeled community structures within MuSW50 or MuSW100. We observed approximately similar clusters in total or BrdU-labeled community structures upon analyzing each treatment condition in duplicate, and, thus, randomly selected samples for a MiSeq ana­lysis.

### Diversities and community structures of total and BrdU-labeled microbes by the Miseq ana­lysis

The number of non-chimeric reads obtained in the present study per sample ranged between 38,839 and 119,950 (Table S2). The values of observed ASVs, Faith’s phylogenetic diversity, Shannon entropy, and Pielou’s evenness were in the ranges of 102–615, 7.36–41.86, 1.93–7.62, and 0.29–0.84, respectively. All diversity indices showed significant differences between total (T0) and BrdU-labeled microbes in SW, MuSW50, and MuSW100 samples (the Wilcoxon rank-sum test, *P*<0.05); those of BrdU-labeled microbes were significantly lower than total (labeled and unlabeled) bacteria (Table 2). In the BrdU communities of mucus samples, almost 50% of the observed ASVs in every SW, MuSW50, and MuSW100 sample were not detected in the total communities (T0 and T10 samples) (Fig. S5). No significant differences were observed in any diversity index for total or BrdU communities among SW, MuSW50, and MuSW100 samples (the Kruskal-Wallis rank sum test, *P*>0.05), except for Shannon entropies in T10 communities (the Kruskal-Wallis rank sum test, *P*=0.0206). Furthermore, there were no significant difference between MuSW50 and MuSW100 samples, even between T10 communities.

**Fig. 1.**
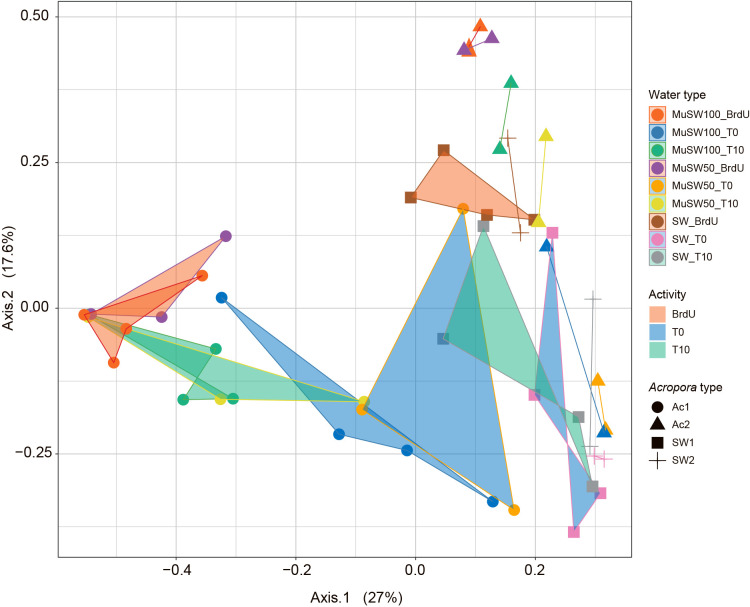
PCoA of community structures for total and BrdU-labeled bacteria in seawater and mucus-containing seawater. Plots and filled areas with distinct colors indicate the water type and total or BrdU-labeled bacteria, respectively. The plot shape indicates seawater, *Acropora* AC1, or AC2.

PCoA showed that changes in the community structures of total microbes (T0 and T10 samples) were greater than in BrdU-labeled microbes (BrdU samples) (Fig. 1). Furthermore, community structures significantly differed between total (T0 and T10) and BrdU-labeled microbes (PERMANOVA, *P*=0.0001) (Table S3). The microbial community structures of mucus-containing seawater from AC1 and AC2 corals significantly differed from those of only seawater (PERMANOVA, *P*=0.0001), along with total (T0 and T10) and BrdU-labeled samples (PERMANOVA, *P*=0.0192). Since no significant differences were observed in alpha or beta diversity between the community structures of MuSW50 and MuSW100 samples, we selected MuSW50 and MuSW100 as a combined sample for box plots or only MuSW100 samples for the PICRUSt2 ana­lysis.

### Differences in microbial community structures between seawater and mucus-containing seawater by the MiSeq ana­lysis

**Fig. 2.**
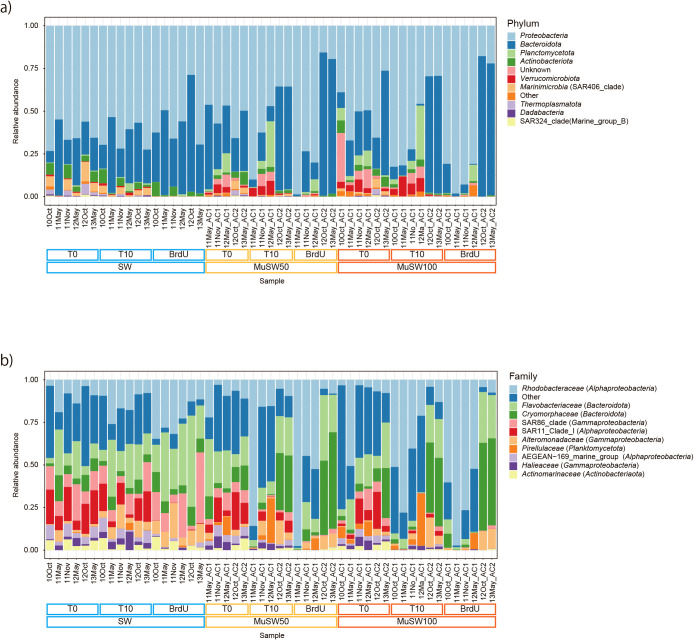
Relative abundance of bacterial taxa at phylum and family levels. Abundance at the a) phylum and b) family levels. Bacterial taxa below the top ten most abundant groups are summarized as “Other”.

**Fig. 3.**
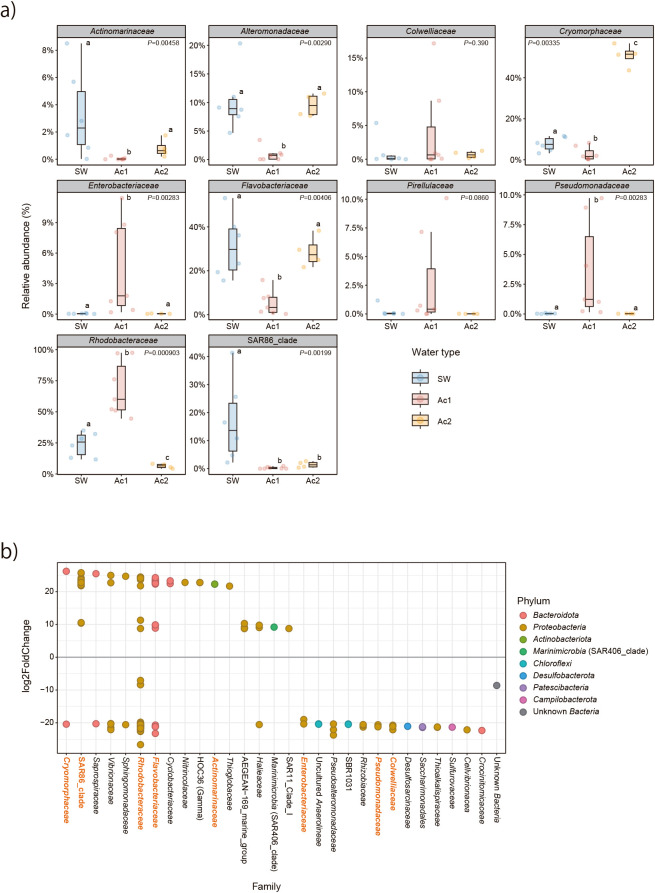
Bacterial taxa of BrdU-labeled communities at the family level in seawater. a) Relative abundance of the top ten bacterial taxa of BrdU-labeled communities at the family level in SW and AC1 and AC2 mucus (MuSW50 and MuSW100). *P* values show the Kruskal-Wallis rank sum test and different letters indicate significant differences between water types (SW, AC1, and AC2) with *P*<0.05 using the Wilcoxon signed-rank test with a Benjamini-Hochberg correction. b) ASVs of BrdU-labeled communities at the family level in SW and MuSW100 samples. ASVs with log2FoldChange>|1| and adjusted *P*<0.01 according to a DESeq2 ana­lysis are shown. Positive and negative values of log2FoldChange indicate significantly abundant ASVs in SW and MuSW100 samples, respectively. The bacterial families shown in red indicate the top ten bacterial taxa at the family level (Fig. 2).

The relative abundance of microbial taxa at the phylum and family levels is shown in Fig. 2, S6, and S7. At the phylum level, *Proteobacteria* and *Bacteroidota* were the dominant taxa in all samples, including SW, MuSW50, and MuSW100; their average values were 55.1 and 26.3% (T0‍ ‍samples), 58.3 and 27.6% (T10 samples), and 59.5 and‍ ‍36.8% (BrdU samples), respectively (Fig. 2a and S6).‍ ‍*Planktomycetota*, *Verrucomicrobiota*, *Marinimicrobia* (SAR406 clade), and unknown Bacteria accounted for more than 10% of the total taxa in some samples (Fig. 2a). Archaea were relatively abundant in SW samples, in the range of 0.006–7.0% (Fig. S7a). In all samples, three bacterial taxa were abundant in both total and BrdU-labeled communities at the class level (Fig. S7b): *Alphaproteobacteria* (avg. 37.2%), *Bacteroidia* (30.2%), and *Gammaproteobacteria* (20.4%). At the family level, *Rhodobacteraceae* (class‍ ‍*Alphaproteobacteria*), *Flavobacteriaceae* (class *Bacteroidia*), and *Cryomorphaceae* (class *Bacteroidia*) were abundant in all samples; average relative abundance values were 11.0, 25.1, and 38.1% (T0 communities); 15.2, 13.5, and 20.0% (T10 communities); and 7.9, 11.1, and 15.8% (BrdU communities), respectively (Fig. 2b). The SAR11 clade (class *Alphaproteobacteria*) was only abundant in T0 and T10 communities, with relative abundance values of 11.1 and 5% on average, respectively. In comparison with SW, the top ten most abundant families in BrdU-labeled bacteria varied with the sample treatment and the mucus source (AC1 or AC2) (Kruskal-Wallis rank sum test, *P*<0.01) (Fig. 3a). The abundance of SAR86 clade (class *Gammaproteobacteria*) bacteria was the highest in SW samples (the Wilcoxon signed-rank test, adjusted *P*<0.05), whereas *Enterobacteriaceae*, *Pseudomonadaceae* (class *Gammaproteobacteria*), and *Rhodobacteraceae* were predominant in AC1 mucus samples and *Cryomorphaceae* species in AC2 samples (the Wilcoxon signed-rank test, adjusted *P*<0.05). *Actinomarinaceae* (class *Actinomycetes*), *Alteromonadaceae* (class *Gammaproteobacteria*), *Cryomorphaceae*, and *Flavobacteriaceae* were less abundant in AC1 mucus (the Wilcoxon signed-rank test, adjusted *P*<0.05) than in SW and AC2 mucus. The DESeq2 ana­lysis also clearly showed differential ASV abundance between communities of BrdU-labeled bacteria in SW and MuSW100 samples (Fig. 3b). Some bacterial families, such as *Rhodobacteraceae* and *Cryomorphaceae*, which were significantly abundant (adjusted *P*<0.01) in mucus-containing seawater at the family level (Fig. 3a), were found to be divided into two groups: predominant species in seawater and predominant species in mucus-containing seawater (Fig. 3b).

### Functional differences in microbial communities between seawater and mucus-containing seawater

According to PICRUSt2 functional predictions, alpha diversities assessed by both EC numbers and MetaCyc pathway databases did not significantly differ between total (T0) and BrdU-labeled microbes in SW, MuSW50, or MuSW100 samples, except for observed functions in mucus-containing samples (the Wilcoxon rank-sum test, *P*<0.01) (Table S4). In BrdU-labeled communities, no significant differences were observed between the diversity indices of SW, MuSW50, and MuSW100 samples (the Kruskal-Wallis test, *P*>0.05). A total of 1,939 of enzyme occurrences, which refers to the frequency at which a specific enzyme appears and may be used to indicate the amount of the enzyme present in each SW and MuSW100 sample, were predicted by PICRUSt2 in BrdU-labeled communities from SW and MuSW100 samples. Significantly more enzyme occurrences were detected in MuSW100 samples (a total of 368) than in SW samples (a total of 118) by the DESeq2 ana­lysis (adjusted *P*<0.01) (Table 3). Among 1,939 enzyme occurrences, approximately 19% were significantly increased in MuSW100 samples, compared to 6% in SW samples. Regarding MetaCyc pathways predicted by PICRUSt2, 382 pathways in the BrdU-labeled communities of SW and MuSW100 samples were observed. Similar to the results obtained on enzyme occurrences, the number of significant pathways was higher in MuSW100 samples than in SW samples (adjusted *P*<0.01) (Table 4). Among 382 pathways, approximately 15% were significantly increased in MuSW100 samples, compared with 5% in SW samples. Pathways associated with amines, amino acids, aromatic compounds, carbohydrates, carboxylates, their metabolites, and inorganic nutrients were abundant in MuSW100 samples.

## Discussion

Using the BrdU labeling approach, we identified previously undetected families of AGB using conventional methods that contribute to coral holobiont regulation and material cycles in surrounding seawater. BrdU is a thymidine analog incorporated by active bacterial DNA synthesis that may be detected with specific antibodies (Steward and Azam, 1999; Hamasaki *et al.*, 2004). The substrate to be labeled with the antibody may vary depending on the purpose of the ana­lysis, and a phylogenetic ana­lysis of AGB in soil and marine environments has been conducted using an immunocapture technique with antibody-conjugated magnetic beads. Previous studies demonstrated that community structures significantly differed between total bacteria and AGB (Taniguchi *et al.*, 2011; Taniguchi and Eguchi, 2020), which is consistent with the present results showing that the observed ASVs were less abundant in actively growing microbes than in total microbes (Table 2). Similar findings were obtained in studies using an identical BrdU approach; however, these studies were not conducted in a coral environment and did not use MiSeq sequencing (Hamasaki *et al.*, 2007; Taniguchi *et al.*, 2015). One possible reason why bacterial species not found in the total bacterial community were detected as AGB species may be the percentage of AGB DNA to the total DNA content in samples. For example, total DNA includes genetic material from dormant and dead bacteria that do not markedly contribute to material cycles. Using the BrdU approach, Tada *et al.* (2009) reported that approximately 20% of total bacteria actively grow throughout the year, even in a eutrophic coastal environment. DNA derived from these AGB species represents a small percentage of total DNA, but a higher percentage of BrdU-labeled DNA, leading to apparent differences in community structures. A potential limitation of the thymidine method is that a small fraction of AGB cannot incorporate BrdU; however, the method generally allows for the detection of a wide range of bacteria without any phylogenetic bias (Hamasaki *et al.*, 2007). Hellman *et al.* (2011) also showed that taxonomically diverse bacteria representing all the dominant phyla incorporated BrdU. Additionally, using metagenomics, Mou *et al.* (2008) demonstrated that a wide range of bacterial taxa in coastal oceans incorporate BrdU. Archaea were detected in some of the seawater samples in the present study, but were mostly present in the unlabeled fraction (Fig. S5a). Since our knowledge of the incorporation ability of BrdU by archaea is highly limited (Dinasquet *et al.*, 2017; Delpech *et al.*, 2018), interpretations for archaea need to be made with caution.

We observed high variability in the community structures of total bacteria, whereas AGB community structures showed fewer variations in both mucus-containing seawater samples and seawater-only samples (Fig. 1). This result suggests that specific bacteria occur as AGB in certain seasons, namely, spring and autumn in the present study. Previous studies reported that bacterial community structures occur annually with repeatable temporal patterns (Fuhrman *et al.*, 2006; Chow *et al.*, 2013) and that similar structures form between spring and autumn (Mestre *et al.*, 2020; Taniguchi and Eguchi, 2020). The factors affecting bacterial community structures are environmental and include water temperature, salinity, and inorganic nutrients (Fuhrman *et al.*, 2006; Mestre *et al.*, 2020). Previous studies on the annual repeatability of bacterial community structures were based on total bacteria, whereas the present study showed that the community structures of AGB were more repeatable over spring and autumn than those of total bacteria. Notably, the annual repeatability of the AGB community structure was observed even in mucus-influenced seawater (Fig. 1), suggesting that specific bacteria contribute to and define the material cycle in certain environments.

In the present study, the community structures of not only total bacteria, but also AGB differed between AC1 and AC2 samples (Fig. 1); however, these specimens were both of the genus *Acropora* (Fig. S1). Previous studies showed that bacterial community structures differed between seawater and mucus samples and also among coral species (Lema *et al.*, 2012; Nelson *et al.*, 2013). However, coral species-specific bacterial associations exist despite differences in geographic locations (Carlos *et al.*, 2013). Furthermore, Taniguchi *et al.* (2014) demonstrated that coral mucus enhanced bacterial production in seawater and this effect varied not only with the coral genus, but also within a single species. Increased bacterial production has been attributed to the significant growth enhancement of bacteria originating from seawater rather than coral mucus (Taniguchi *et al.*, 2014; Taniguchi *et al.*, 2015). Moreover, the composition of mucus was found to vary among coral species (Wild *et al.*, 2010). Based on these findings, differences in the community structures of‍ ‍AGB between AC1 and AC2 may have been caused by bacterial growth in seawater due to coral species-specific characteristics.

The results of our DESeq2 ana­lyses showed that AGB taxa may be divided into two groups based on their differential bacterial growth response in seawater (Fig. 3b): seawater specialists that originally grow in seawater, and mucus specialists that grow under the influence of coral mucus. The bacterial preference of coral mucus was also differ­ent‍ ‍between coral AC1 and AC2. *Rhodobacteraceae*, *Enterobacteriaceae*, and *Pseudomonadaceae* bacteria, which were detected as AGB among AC1 mucus (Fig. 3a), have been identified as abundant bacteria associated with a wide range of coral species (Huggett and Apprill, 2019). A previous study reported that *Rhodobacteraceae* bacteria prefer osmolytes in coral mucus (Luo *et al.*, 2021). These bacteria may have a high preference for mucus as mucus specialists, even after it is released into the surrounding environment. On the other hand, *Alteromonadaceae* and *Flavobacteriaceae* bacteria have often been found in coral mucus (Huggett and Apprill, 2019), but may be inhibited or not favored as growth substrates by coral AC1 mucus (Fig. 3a). Another study identified *Alteromonadaceae* species as the dominant bacteria in *Acropora* mucus-supplemented seawater; our contradictory results strongly suggest a difference in the bacterial preference of coral mucus, even within the genus *Acropora*. *Vibrionaceae* and *Pseudoalteromonadaceae* were detected as AGB with a mucus preference in the present study (Fig. 3b). These bacteria have often been isolated from corals (Huggett and Apprill, 2019), and are known to be associated with coral diseases (Ben-Haim *et al.*, 2003; Shnit-Orland *et al.*, 2012). *Cryomorphaceae* bacteria, which were previously unknown bacteria associated with coral mucus, were the most predominant AGB that responded to coral AC2 mucus (Fig. 3a). *Cryomorphaceae*, along with *Saprospiraceae* (ASV with a mucus preference, Fig. 3b), has been shown to prefer substrates containing alginate, a gelatinous polysaccharide particle (Mitulla *et al.*, 2016). The key component of the polysaccharide produced by stony coral is a type of uronic acid (Naggi *et al.*, 2018), and *Cryomorphaceae* bacteria may have been actively growing using polysaccharides derived from coral mucus. Future studies are needed to establish why the growth patterns of mucus-influenced bacteria, such as *Cryomorphaceae*, differ within the same *Acropora* coral; however, in consideration of the heterogeneity of coral assemblages, these results strongly suggest that unexpectedly diverse and complex bacterial community structures formed in the area where this study was conducted as well as in reef environments.

The AGB community in mucus-influenced seawater showed highly distinct diverse enzymes and pathways from those in seawater (Table 3 and 4) and its functions varied with changes in the composition of AGB. It is important to note that these functional diversities were predicted despite the lack of significant differences in species diversity between these two communities. In the present study, diverse functions appeared to reflect the contents of coral mucus, which include various materials, such as amines, amino acids, sugars, glycolic acids, aromatic compounds, and inorganic nutrients, including nitrate, ammonia, and phosphate (Wild *et al.*, 2005; Nakajima *et al.*, 2009, 2015; Tanaka *et al.*, 2009). A previous study demonstrated that the contents of coral mucus appeared to vary not only due to environmental stress, such as an increasing water temperature (Lee *et al.*, 2016) and irradiation (Crossland, 1987; Kuwahara *et al.*, 2010), but also between different coral species (Wild *et al.*, 2010). Lee *et al.* (2016) showed that thermal stress changed the mucus sugar content, which affected the mucus-associated bacterial community. To gain a more comprehensive understanding of bacterial responses after coral releases mucus into surrounding seawater, the combined effects of released mucus and anticipated stressors on reef holobiont communities need to be examined because changes in both mucus contents and associated bacterial communities may have an impact on the surrounding environment. In consideration of the significance of clustering diverse corals in this farming area (Hata *et al.*, 2013), this environment is likely to exhibit a stronger response to organic loading by aquaculture activity because the community structure of diverse microbes has been fostered by coral mucus to accommodate various organic matter.

The present study demonstrated the effects of coral mucus on bacterial community structures and functions. Moreover, the results obtained showed that bacterial taxa contained species with different preferences for coral mucus: mucus specialists, seawater specialists, and generalists. The presence of corals in this fish farming area is associated with more complex bacterial dynamics, both in terms of species and function, than in areas lacking corals. Since microbes are involved in the degradation of organic matter, the role of coral mucus in maintaining purifying bacteria needs to be investigated. If the presence of coral is shown to promote the degradation of organic matter, this information may be used to justify the conservation and restoration of coral communities in fishery sites. Since specific microbes, such as *Rhodobacteraceae* and *Cryomorphaceae*, contribute to biogeochemical cycles, further studies are needed to investigate the role of these bacteria in the degradation processes of organic matter in this environment. Moreover, in consideration of the various forms of coral mucus aggregates (Naumann *et al.*, 2009; Bythell and Wild, 2011), it is plausible to assume that coral mucus markedly affects bacterial dynamics, even at the microbial scale; this effect may be more extensive than previously thought. Further ana­lyses from a microscale perspective (Azam, 1998; Stocker, 2012) are needed to examine the real effects of coral mucus on the surrounding environment. The present study revealed the growth responses of specific bacterial taxa to the release of coral mucus, thereby demonstrating the higher diversity of predicted enzymes and degradation pathways than those in seawater. These results suggest that corals foster bacterial communities with diverse functions, which, in turn, contribute to the redundancy of the entire microbial community and the ecological resilience of this fish farming area.

## Citation

Taniguchi, A., Kuroyanagi, Y., Aoki, R., and Eguchi, M. (2023) Community Structure and Predicted Functions of Actively Growing Bacteria Responsive to Released Coral Mucus in Surrounding Seawater. *Microbes Environ ***38**: ME23024.

https://doi.org/10.1264/jsme2.ME23024

## Supplementary Material

Supplementary Material

## Figures and Tables

**Table 1.** Total organic carbon (TOC) concentrations in the present study.

Date	Water type	Bottle #1			Bottle #2	
		TOC concentration (mg L^–1^)	% of mucus-C^a^		TOC concentration (mg L^–1^)	% of mucus-C^a^
2010-Oct	SW	1.349±0.008	—		no sample	
	MuSW50	no sample			no sample	
	MuSW100	2.240±0.031	66.0		2.364±0.009	75.2^b^
2011-May	SW	0.860±0.011	—		0.885±0.006	—
	MuSW50	1.309±0.002	52.2		1.461±0.018	65.1
	MuSW100	2.006±0.005	133.3		2.073±0.009	134.2
2011-Nov	SW	0.962±0.013	—		0.922±0.008	—
	MuSW50	1.284±0.025	33.5		1.250±0.009	35.6
	MuSW100	1.629±0.023	69.3		1.522±0.025	65.1
2012-May	SW	1.085±0.016	—		1.047±0.020	—
	MuSW50	1.777±0.015	63.8		1.787±0.024	70.7
	MuSW100	2.771±0.035	155.4		2.321±0.026	121.7
2012-Oct	SW	0.844±0.011	—		0.819±0.009	—
	MuSW50	0.868±0.006	2.8		0.876±0.004	7.0
	MuSW100	0.931±0.008	10.3		0.936±0.010	14.3
2013-May	SW	0.792±0.012	—		0.854±0.014	—
	MuSW50	0.934±0.017	17.9		0.941±0.008	10.2
	MuSW100	1.147±0.010	44.8		1.179±0.003	38.1

All values were significantly different among the three water type (Tukey’s HSD test, *P*<0.05). ^a^ Calculated as follows: ([MuSW50 or MuSW100 TOC concentration]–SW TOC concentration)/(SW TOC concentration)×100 ^b^ The value calculated from the TOC concentration of SW in bottle #1.

**Table 2.** Alpha diversities of bacteria in seawater supplemented with and without mucus (ave±SD).

Water type	Activity	Observed ASV	Faith pd	Shannon entropy	Pielou’s evenness
SW	T0	456±102	26.5±6.49	7.07±0.65	0.80±0.05
	T10	433±77	25.5±3.83	6.70±0.55	0.77±0.05
	BrdU	214±36	12.9±1.79	4.68±0.61	0.61±0.08
MuSW50	T0	473±95	32.2±4.59	6.91±0.60	0.78±0.05
	T10	343±92	23.5±5.28	5.45±1.37	0.65±0.15
	BrdU	178±53	13.4±5.25	3.82±1.46	0.51±0.18
MuSW100	T0	438±149	33.5±5.46	6.51±0.89	0.75±0.08
	T10	308±77	23.0±5.93	5.17±1.08	0.63±0.11
	BrdU	160±36	11.7±2.52	3.93±1.18	0.54±0.15

**Table 3.** Predicted enzyme occurrence by PICRUSt2 in actively growing bacteria. The number of enzymes that significantly differ as per the DESeq2 ana­lysis in each water type (log2FoldChange>|1| and adjusted *P*<0.01) is shown.

Class	Enzyme type	SW	MuSW100
EC1	Oxidoreductases	24	93
EC2	Transferases	45	96
EC3	Hydrolases	24	96
EC4	Lyases	10	45
EC5	Isomerases	11	20
EC6	Ligases	4	18
Total		118	368

**Table 4.** List of predicted pathways by PICRUSt2 in actively growing bacteria. The number of pathways that significantly differ as per the DESeq2 ana­lysis in each water type (log2FoldChange>|1| and adjusted *P*<0.01) is shown.

Pathway	SW	MuSW100
Alcohol Degradation	0	1
Aldehyde Degradation	0	1
Amine and Polyamine Biosynthesis	0	2
Amine and Polyamine Degradation	2	4
Amino Acid Biosynthesis	0	2
Amino Acid Degradation	1	4
Aromatic Compound Degradation	0	8
Butanediol Biosynthesis	0	2
C1 Compound Utilization and Assimilation	0	1
Carbohydrate Biosynthesis	2	4
Carbohydrate Degradation	0	5
Carboxylate Degradation	0	5
Cell Structure Biosynthesis	0	4
Cofactor, Carrier, and Vitamin Biosynthesis	2	3
Degradation pathways for the two dicarboxylic acid sugars	0	1
Fatty Acid and Lipid Biosynthesis	3	0
Fermentation	0	1
Generation of Precursor Metabolites and Energy	1	0
Inorganic Nutrient Metabolism	0	4
Nucleic Acid Processing	1	0
Nucleoside and Nucleotide Biosynthesis	3	0
Nucleoside and Nucleotide Degradation	2	0
Secondary Metabolite Biosynthesis	0	2
Secondary Metabolite Degradation	0	1
Tetrapyrrole Biosynthesis	1	0
TCA cycle VII	0	1
Total	18	56
