# Supplementary material for: Community Structure and Predicted Functions of Actively Growing Bacteria Responsive to Released Coral Mucus in Surrounding Seawater
Source: Microbes Environ. 2023 Sep 14;38(3):ME23024. doi: 10.1264/jsme2.ME23024 (PMC10522842; doi:10.1264/jsme2.ME23024)
Supplement: Supplementary file 1 — Supplementary Material [file 38_23024_s1.pdf]

a) b)

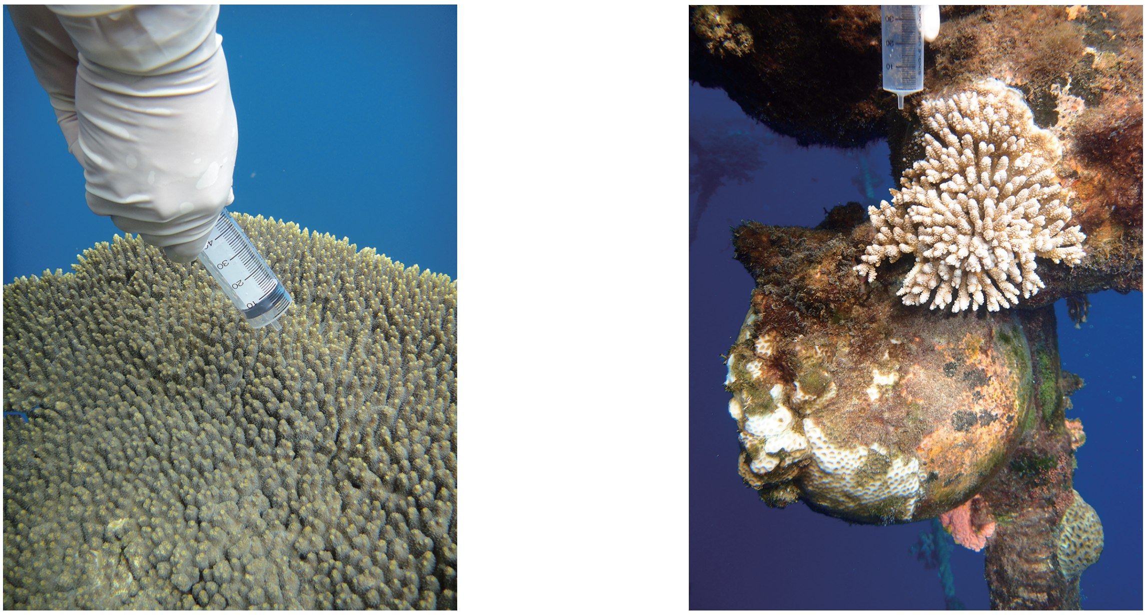

Fig. S1 Corals used in this study.

a) *Acropora* sp. AC1 for October 2010, May and November 2011, and May 2012, b) *Acropora* sp. AC2 for October 2012, and May 2013.

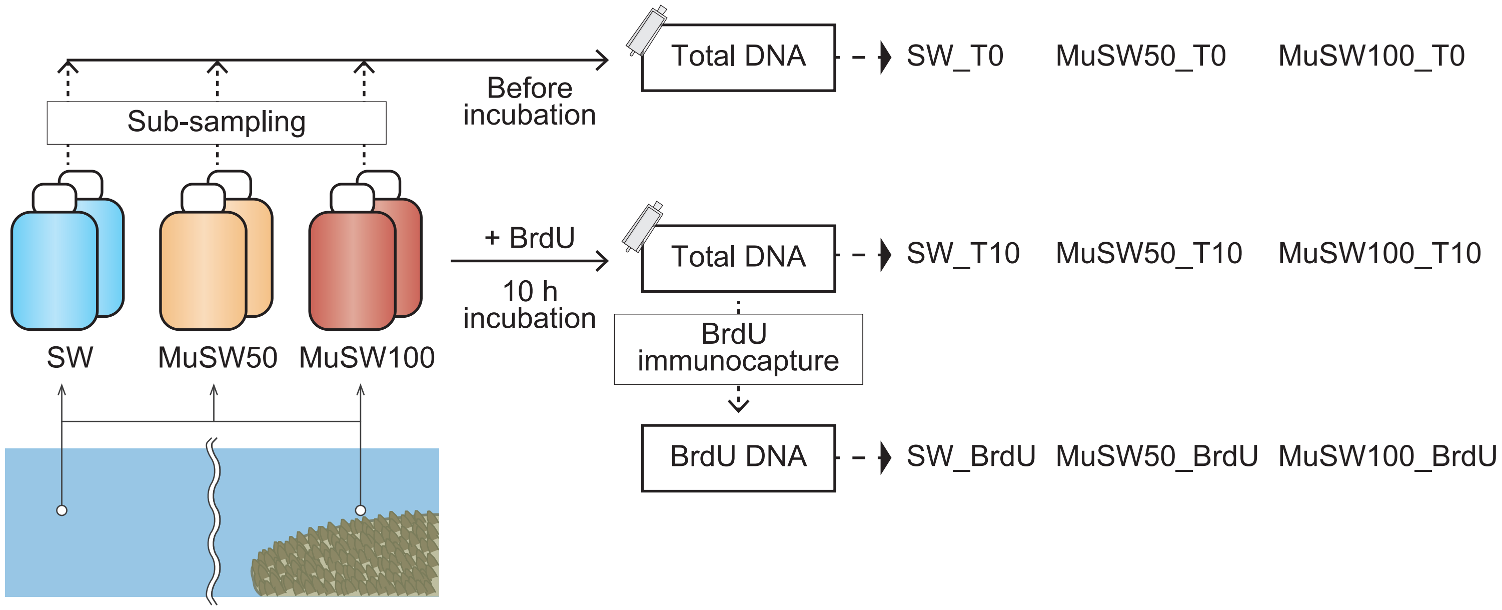

Fig. S2 Simplified experimental design and DNA sample ID.

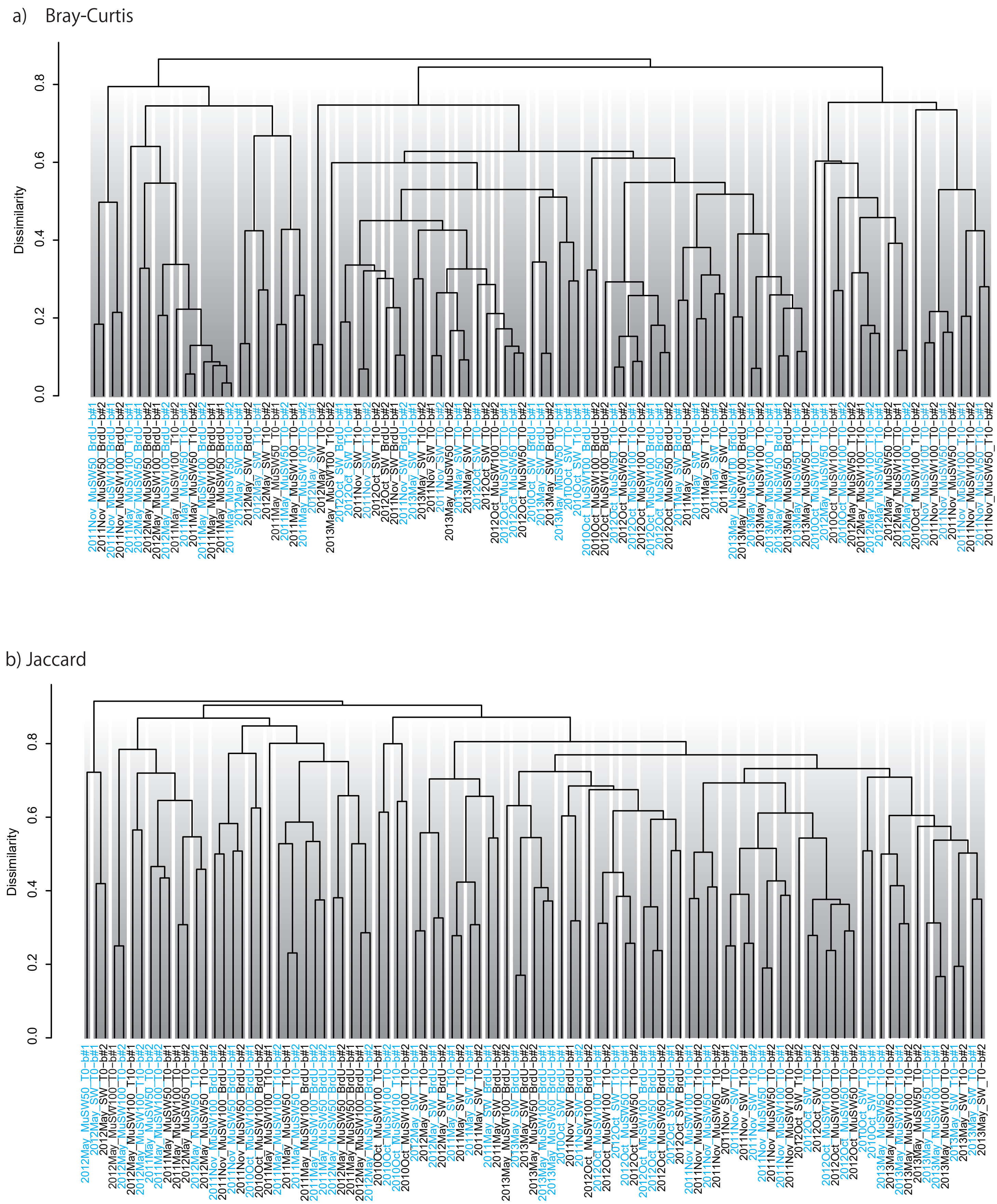

Fig. S3 Cluster analysis of ARISA peak patterns of total and BrdU-labeled communities.

The clusters were based on Bray-Curtis dissimilaritiy (a) and Jaccard index (b). Each gray box indicates distinguishable clusters (SIMPROF, *P*<0.05). Sample used for MiSeq analysis are shown in blue.

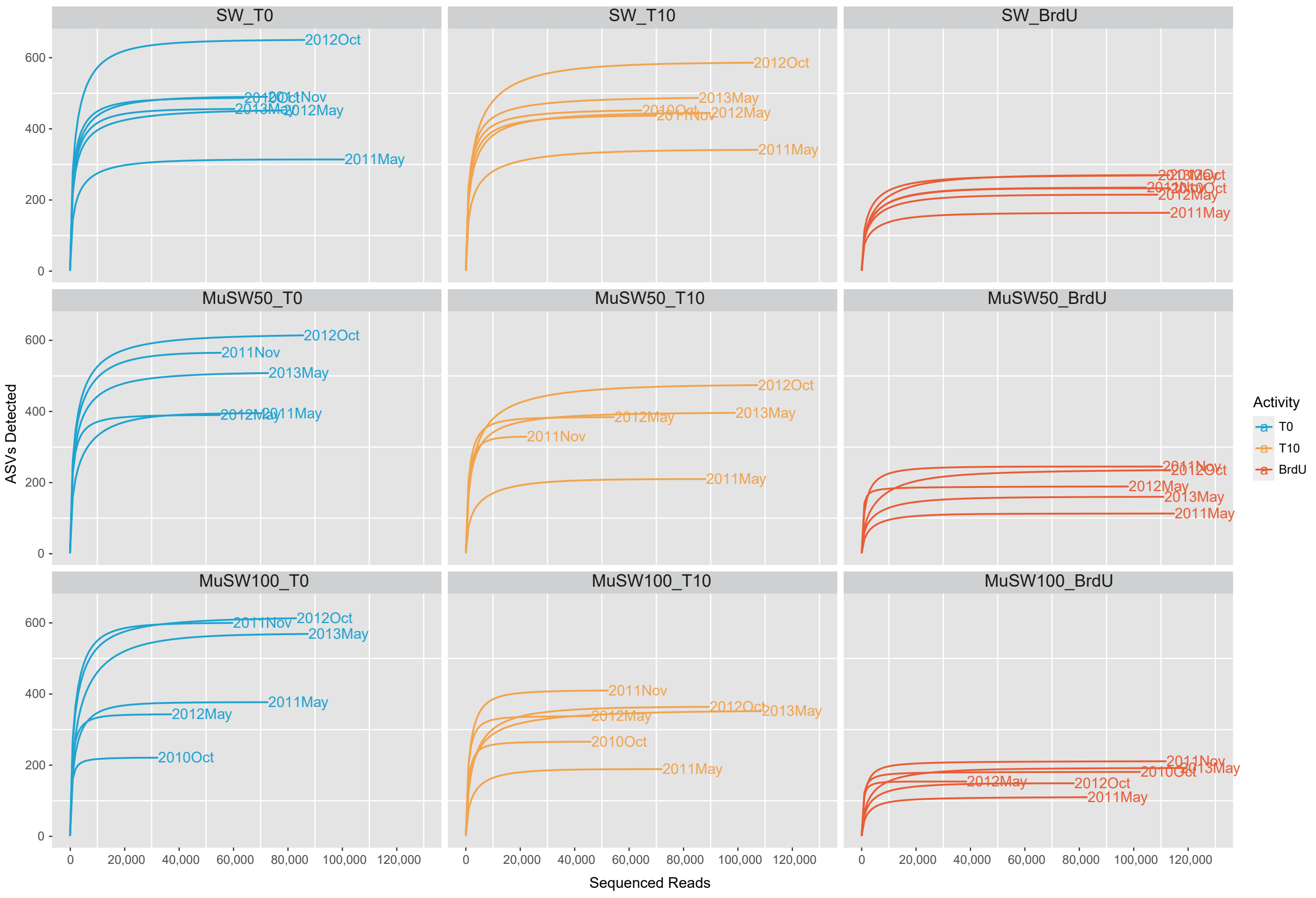

Fig. S4 Rarefaction curve of ASVs detected in this study.

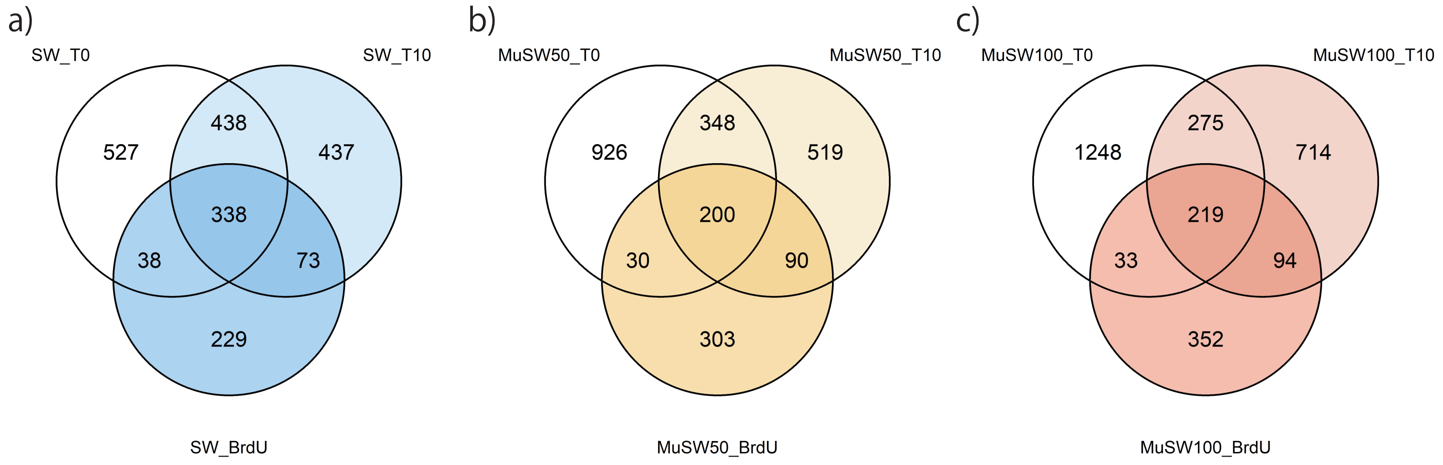

Fig. S5 Venn diagrams of observed features in T0, T10 and BrdU samples of either SW (a), MuSW50 (b), or MuSW100 (c). The number of observed features for each water type is the sum set of values in Table 2.

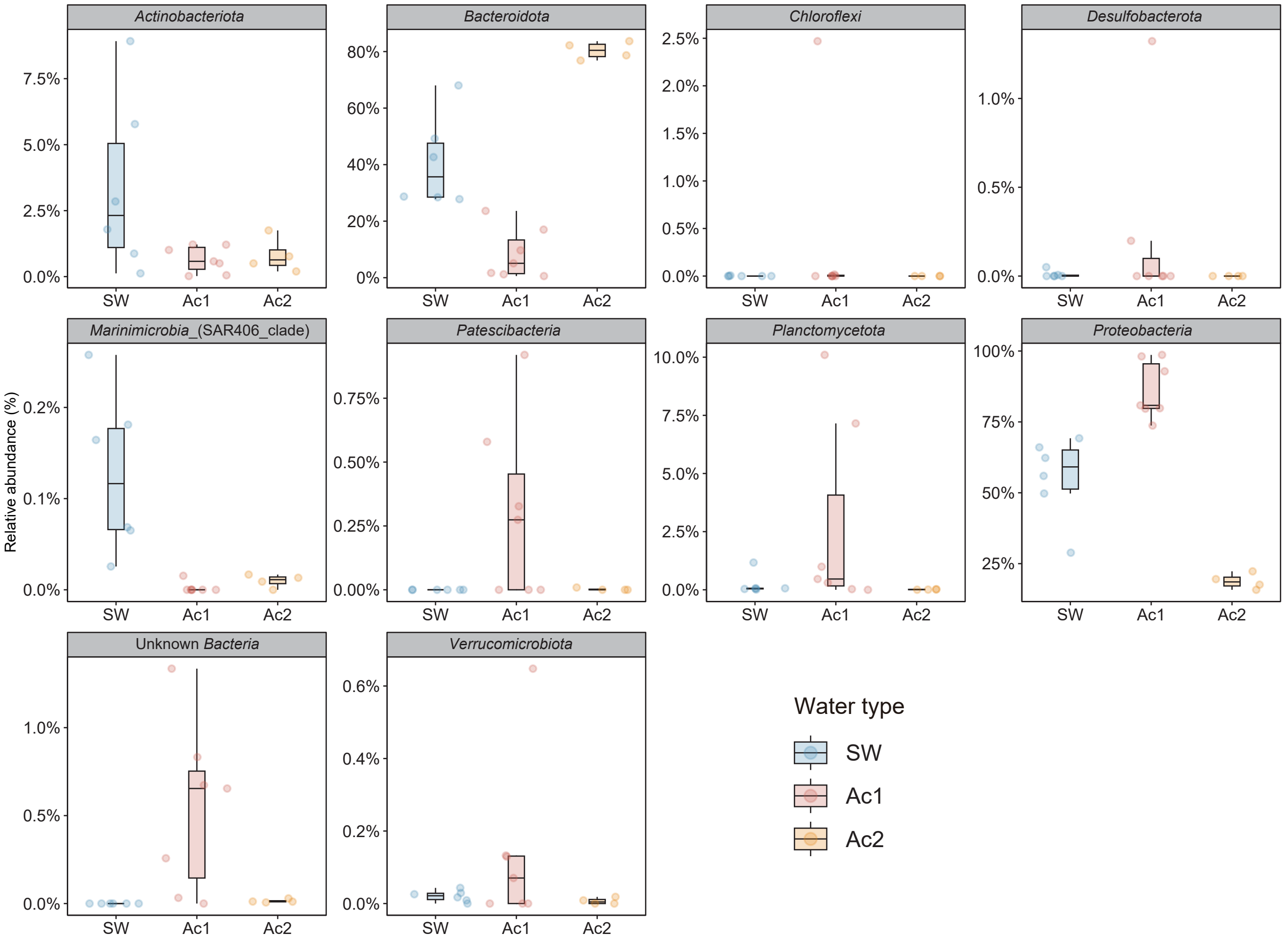

Fig. S6 Relative abundance of the top ten bacterial taxa of BrdU-labeled communities at the phylum level in SW, and AC1 and AC2 mucus (MuSW50 and MuSW100).

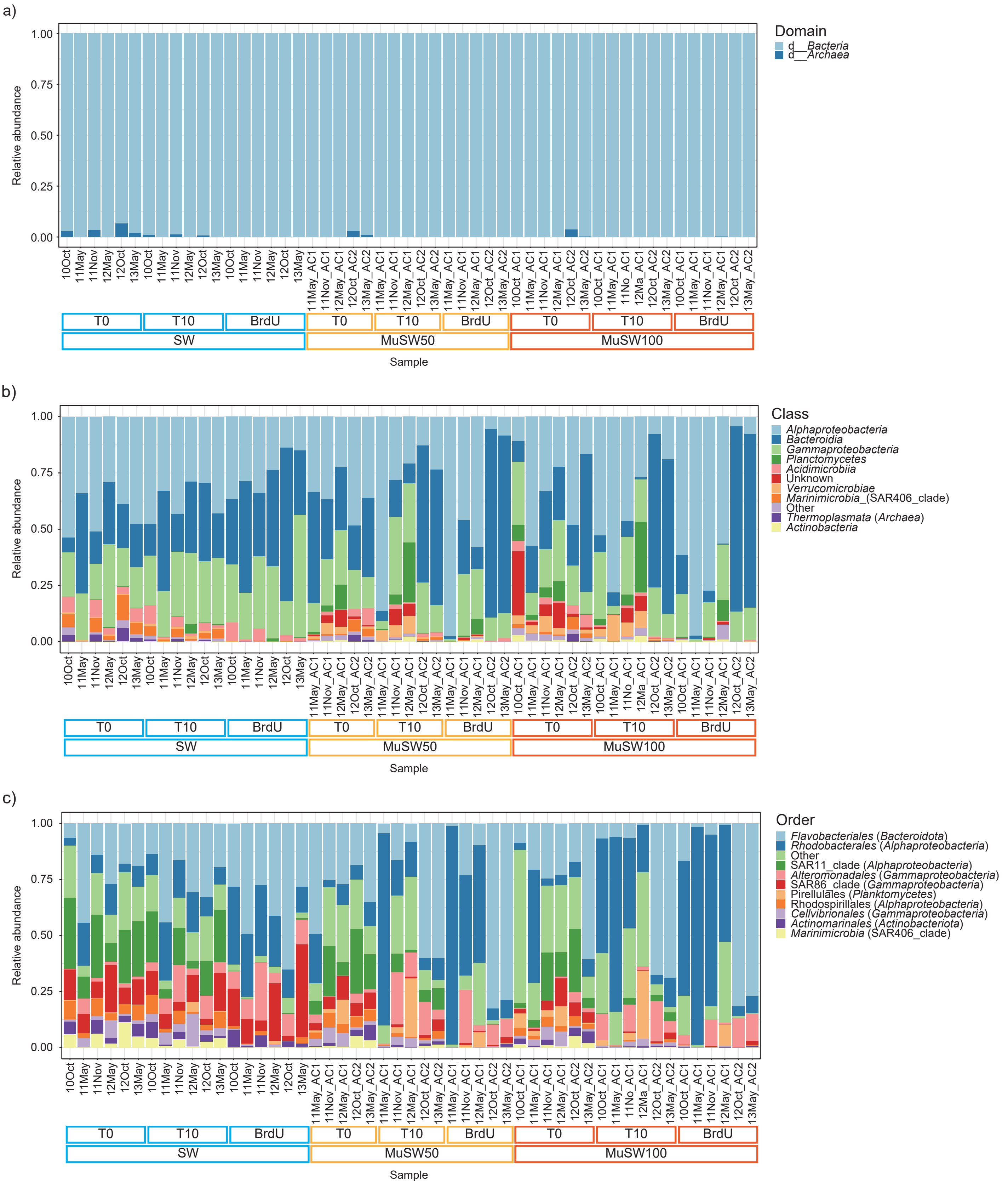

Fig. S7 Relative abundance of bacterial taxa at the domain (a), class (b), and order (c) levels. Bacterial taxa below the top ten abundance were summarized as Other.
